# Single-Cell Transcriptomic Analysis Reveals Macrophage–Tumor Crosstalk in Hepatocellular Carcinoma

**DOI:** 10.3389/fimmu.2022.955390

**Published:** 2022-07-25

**Authors:** Yunhe Liu, Lin Zhang, Xinyi Ju, Sheng Wang, Jingbo Qie

**Affiliations:** ^1^ Department of Clinical Laboratory Medicine, Shanghai Fifth People’s Hospital, Fudan University and Institute of Biomedical Sciences, Fudan University, Shanghai, China; ^2^ Center of Emergency and Intensive Care Unit, Jinshan Hospital, Fudan University, Shanghai, China; ^3^ STEM Club, Pinetree Secondary School, British Columbia, Canada

**Keywords:** hepatocellular carcinoma, crosstalk, single-cell, CellPhoneDB, interaction

## Abstract

As one of the most malignant cancer, hepatocellular carcinoma (HCC) has a complex ecosystem featured by high heterogeneity. Cell crosstalk is demonstrated to be critical for HCC development. However, the cell communication orchestration in HCC remains largely unknown. Here, by analyzing the single-cell transcriptomes of the primary tumor tissues (n = 10) and tumor-adjacent tissues (n = 8) derived from 10 patients with HCC, we found that the proportions of plasmacytoid dendritic cells (pDCs) and natural killer (NK) cells were reduced and that the proportion of macrophages was increased in the immune component of the primary tumor, compared with those in the tumor-adjacent tissue. Furthermore, we found widespread communication between macrophage populations and other cell types, and this communication was remarkably strengthened in the primary tumor, especially with HCC malignant cells. In addition, the SPP1–CD44 axis was identified as a unique interaction between macrophages and HCC malignant cells. Our comprehensive portrait of cell communication patterns over the HCC ecosystem reveals further insights into immune infiltration.

## Introduction

As one of the most malignant cancer, hepatocellular carcinoma (HCC) has a complex ecosystem featured by high heterogeneity (1). A thorough exploration of hepatocarcinogenesis could contribute to the illustration of mechanisms participating in HCC development and help in the exploration of effective treatment strategies for HCC.

HCC is a complex ecosystem featured by complex cell–cell communications among different heterogeneous cell types (2). The development of tumor cells for coordinated cell crosstalk patterns in the unique ecosystem. Compared with other types of cancer, HCC is strongly dependent on the immune cells’ account and activity in its ecosystem (3). Thus, comprehensively exploring the states of the immune cells in the HCC ecosystem or HCC microenvironment becomes vital for immunotherapeutic strategies as well as the identification of new biomarkers of HCC. The tumor microenvironment (TME) consists of heterogeneous immune component mixtures (4, 5). With the development of HCC, a large number of immune cells transfer to the liver, interact with stromal cells, and establish an active immune environment, which can affect the progress of HCC. Therefore, it is of great significance to illustrate the composition and state of immune cells during hepatocarcinogenesis.

Due to the high heterogeneity of HCC, it remains mostly intractable to existing treatments (6). Most of the current omics technologies take the tissue blocks as the research object, resulting in the loss of important data on cell–cell communication (3). Single-cell RNA sequencing (scRNA-seq), as an emerging omics technology, can be used to study single-cell expression patterns in bulk pathological tissue, making it possible to study the relationship between the microenvironment crosstalk and the state of the diseases. Increasing studies have reported the state of cell–cell communication in TME using scRNA-seq (7–12). However, most studies focused on tumor cells’ heterogeneity in HCC (13–16). A comprehensive depiction of cell crosstalk (or cell–cell communication) in HCC remains lacking.

In our study, we explored the cell crosstalk and TME in HCC by systematically analyzing the scRNA-seq dataset, GSE149614. It was demonstrated that the proportions of plasmacytoid dendritic cells (pDCs) and natural killer (NK) cells were reduced and that the proportion of macrophages was increased in the immune component of HCC, compared with those in tumor-adjacent tissues. Furthermore, we found macrophages widely communicated with the other types of cells, and this communication was remarkably strengthened in HCC, especially with HCC malignant cells. In addition, the SPP1–CD44 axis was identified as a unique interaction between macrophages and HCC malignant cells. Our findings highlighted the dynamic immune response alteration of macrophages in HCC, suggesting novel immunotherapeutic strategies against this disease.

## Materials and Methods

### Single-Cell RNA Sequencing Data Processing

The scRNA-Seq Dataset of GSE149614 was obtained from the Gene Expression Omnibus (GEO) database. Cells that have <500 or >5,000 detected genes and contain mitochondrial genome >5% of total unique molecular identifiers (UMIs) were deleted. A total of 23,225 tumor-adjacent tissue cells and 22,677 HCC tissue cells were included. Considering the processing operation differences between the adjacent-tumor tissue and the tumor tissue before sequencing, the integration function based on the mutual nearest neighbors (MNNs) (17) algorithm provided by Seurat software (version 3.1.1) was used to remove the batch effect between the two datasets. Specifically, the adjacent-tumor data were used as the reference dataset, the FindAnchors function was used to find the nearest neighbor between the two datasets, and the IntegrateData function was used to remove the batch differences between the two datasets and merge them. The use of the Seurat software was continued for clustering operations. First, the merged data were normalized, and the top 2,000 variable genes were hunted for principal component analysis (PCA) dimensionality reduction (dim = 30). After that, the FindNeighbors function (principle component dim = 30) was used to construct the cell nearest neighbor network, and finally, the FindClusters function was used to perform community-based clustering of cells (Louvain; resolution = 0.5). Cell distribution was visualized by the uniform manifold approximation and projection (UMAP) method. All clusters were manually annotated according to the previous report (18).

### Deferentially Expressed Genes in Specified Cell Types

To analyze the functional alteration of specified cell types, differentially expressed genes (DEGs) were obtained by FindMarkers function in Seurat (18) with fold changes ≥1.25 and *adjusted p* < 0.05. The volcano plot was generated by GraphPad 8.

### Cell Crosstalk Analysis

CellPhoneDB (19) is a public database of receptor–ligand interactions. Here, CellPhoneDB (version 2.1.1) was utilized to explore the crosstalk of cell subtypes in HCC. The calculation of the mean value and p-value was defined by CellPhoneDB (**Table S1**). The correlation intensions between specified cell types were shown as the total mean and the number of interactions.

### Definition of Macrophage Scores

To assign M1/M2 polarization estimates to macrophage cells, Gene Set Variation Analysis (GSVA) was applied using standard settings, as implemented in the GSVA package (20). The gene sets associated with the above functions were described by Azizi et al. (10) (**Table S2**).

### Gene Ontology and Kyoto Encyclopedia of Genes and Genomes Pathway Functional Enrichment Analyses

Gene Ontology (GO) annotation and Kyoto Encyclopedia of Genes and Genomes (KEGG) analyses were performed and visualized using clusterProfiler and ggplot2 R package, respectively (19).

### Protein–Protein Interaction Network Construction

The DEGs were mapped to the STRING database (http://strin g-db.org) to assess protein–protein interaction (PPI) within HCC tissue (21). The PPI network was constructed using Cytoscape software (version 3.6.0).

### Validation of Differential Expression of Selected Genes

To validate the expression of selected genes between tumor-adjacent tissues and HCC tissues, HCC patient’s gene expression data (fragments per kilobase of transcript per million mapped reads (FPKM) processed, n = 421, including 50 normal tissues and 371 HCC tissues) from The Cancer Genome Atlas Liver Hepatocellular Carcinoma (TCGA-LIHC) database (https://xenabrowser.net/datapages/) and the Human Protein Atlas (HPA) database (https://www.proteinatlas.org/, for protein expression) (22) were used in our study.

### Survival Analysis

For survival analysis, the survival data (n = 368) were downloaded from TCGA-LIHC database (https://xenabrowser.net/datapages/). The Kaplan–Meier survival curves were visualized by GraphPad 8, and the survival difference between groups was tested by a log-rank test (23).

## Results

### Landscape of the Cell Composition in Non-Tumor and Tumor Tissues

**Figure 1:**
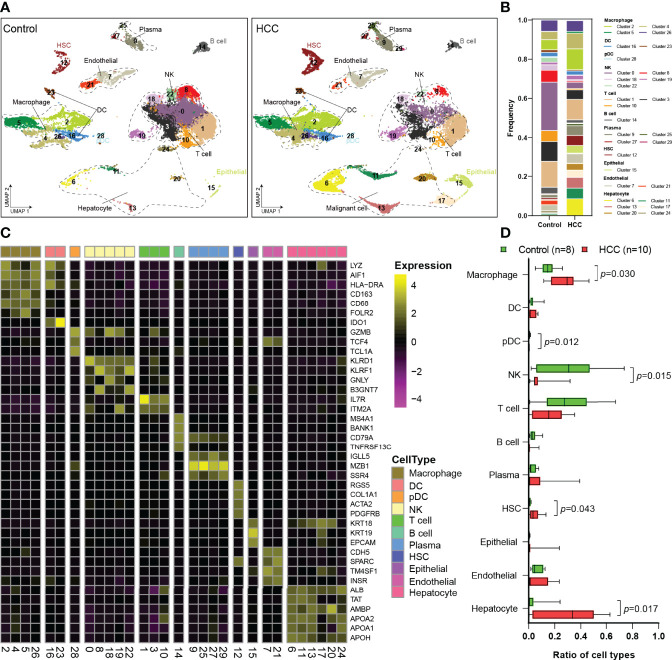
Single-cell atlas of the HCC and tumor-adjacent tissues. **(A)** Overview of the cell clusters based on scRNA-seq data from tumor-adjacent and HCC tissues (UMAP). **(B)** The frequency of cells in each cluster. **(C)** Heatmap showing the expression of marker genes in the indicated cell types. The bottom bars label the clusters corresponding to specific cell types, and the number in brackets corresponds to the cluster number in panel **(A, D)** Histogram indicating the proportion of cells. HCC, hepatocellular carcinoma; scRNA-seq, single-cell RNA sequencing; UMAP, uniform manifold approximation and projection.

The scRNA-Seq dataset of GSE149614 was obtained from the GEO database. After data processing, 23,225 cells from tumor-adjacent tissues (control, n = 8) and 22,677 cells from HCC tissues (n = 10) were used for further analysis. To explore the landscape of the cell composition, the classification of cells and identification of marker genes were performed as in a previous study (18). The cells were divided into 30 clusters through UMAP dimensionality reduction (**Figures 1A**, **S1**), and the frequency of cell types is shown in **Figure 1B**.

Then, 11 cell types were identified among the 30 clusters, including 4 types of non-immune cells and 7 types of immune cells (**Figure 1C**). Non-immune cells were mainly composed of endothelial cells (Es; CDH5, SPARC, TM4SF1, and INSR), hepatic stellate cells (HSCs; RGS5, COL1A1, ACTA2, and PDGFRB), apparently normal epithelial cells (KRT18, KRT19, and EPCAM), and hepatocyte or HCC malignant cells (**Figure 1C**). Immune cells primarily consisted of macrophages (LYZ, AIF1, HLA-DRA, CD163, CD68, and FOLR2), DCs (LYZ, AIF1, HLA-DRA, and IDO1), pDCs (GZMB, TCF4, and TCL1A), NK cells (KLRD1, KLRF1, GNLY, and B3GNT7), T cells (IL7R and ITM2A), B cells (MS4A1, BANK1, CD79A, and TNFRSF13C), and plasma cells (IGLL1, MZB1, and SSR4) (**Figure 1C**). Then, the proportions of each cell type were calculated in tumor-adjacent tissues (n = 8) and HCC (n = 10) (**Figure 1D**). We found that the proportions of pDCs and NK cells in tumor tissues were significantly decreased and the proportions of T cells and B cells were also obviously decreased (**Figure 1D**). Consistent with the previous report (2), we found that the proportion of macrophages was significantly increased in our data.

### Differentially Expressed Genes in Specific Cell Types

**Figure 2:**
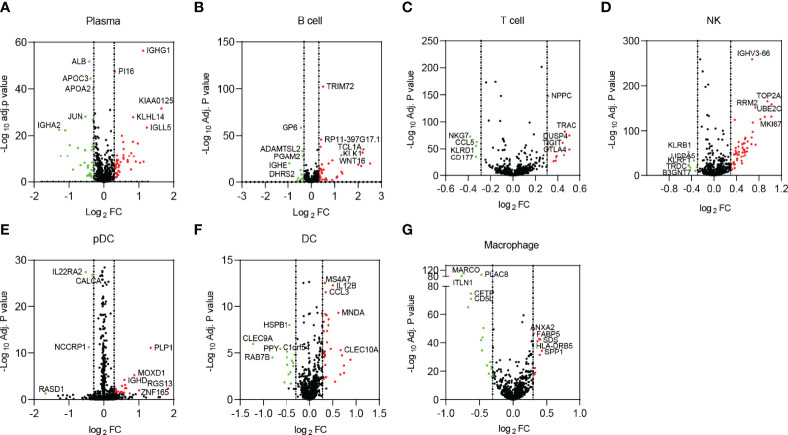
DEGs in immune cells. **(A)** Plasma cells. **(B)** B cells. **(C)** T cells. **(D)** NK cells. **(E)** pDCs. **(F)** DCs. **(G)** Macrophages. The dots in red represent upregulated genes, and the dots in green represent downregulated genes. DEGs, differentially expressed genes; NK, natural killer; pDCs, plasmacytoid dendritic cells.

Next, DEGs of non-immune cells (**Figure S2**) and immune cells (**Figure 2**) between tumor-adjacent tissues and HCC were identified, and the top dysregulated genes were marked. Then, we focused on the immune cells significantly changed in tumor tissues, including NK cells and macrophages.

In NK cells, 54 upregulated and 7 downregulated genes were identified in HCC, most of which had been reported to be involved in the p53 signaling pathway and cell cycles, such as CCNB1, CCNB2, CDK1, PTTG1, PLK1, MAD2L1, GTSE1, and TOP2A. These genes and related pathways were demonstrated to maintain NK cell homeostasis (24). Moreover, some other DEGs, including HSPA1A, HSPA1B, HSPA5, HSPA6, IFNG, CD8B, and KIR2DL3, were reported to participate in antigen processing and presentation. The above DEGs may affect the nature of NK cells and contribute to the immune escape of HCC.

In macrophages, 25 DEGs were screened out, consisting of 9 upregulated and 16 downregulated. Interestingly, most of the DEGs were membrane proteins or secretory proteins, contributing to immune response. For example, SPP1 (also known as OPN), a glycoprotein secreted by macrophages, was reported to mediate HCC malignant cell–macrophage communication (25). CLEC4E, which is located in the cell membrane of the macrophages, regulates macrophage polarization by enhancing endoplasmic reticulum stress response and inhibiting cholesterol efflux (26).

### Cell–Cell Communication in Hepatocellular Carcinoma

**Figure 3:**
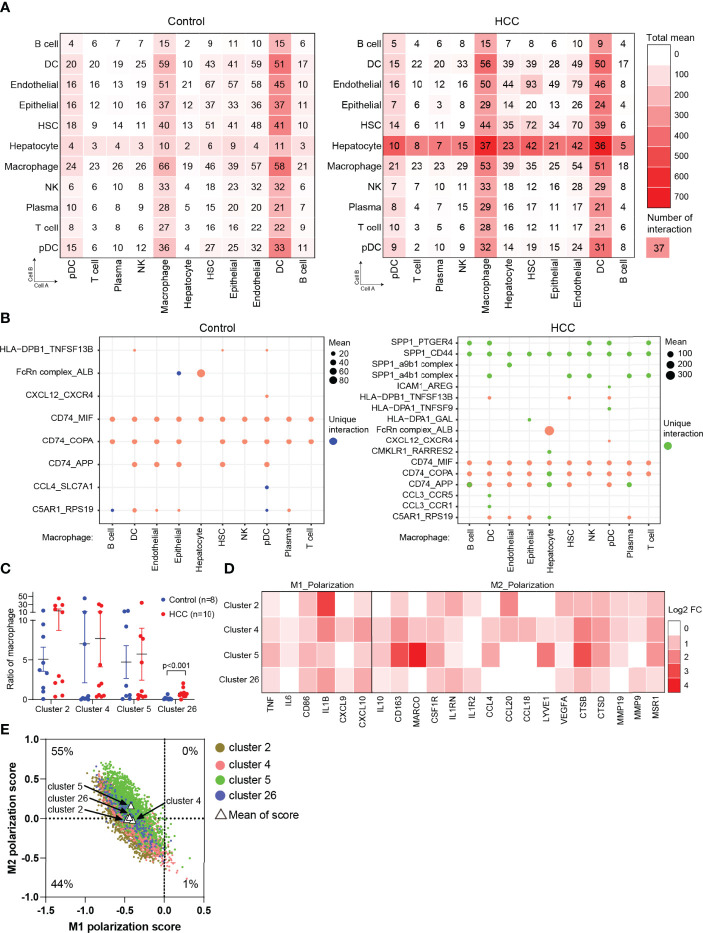
Cell crosstalk in HCC. **(A)** The strength of cell crosstalk shown as total mean and number of interactions. **(B)** Cell crosstalk based on ligand–receptor interaction in the non-tumor and tumor tissues (significant mean >10). **(C)** Ratio of macrophages of clusters 2, 4, 5, and 26 in the individuals. **(D)** The phenotype of macrophages in HCC. **(E)** Scatterplots showing M1 and M2 scores for each color-coded cluster of macrophages. HCC, hepatocellular carcinoma.

To uncover the cellular crosstalk in tumor-adjacent tissues and HCC tissues, the analysis of receptor–ligand interactions was performed through CellPhoneDB (**Table S1**). The correlation intensions between cell A (x-axis) and cell B (y-axis) were shown as the total mean and the number of interactions. The results showed that myeloid-derived cells (macrophages and DCs) widely communicated with the other types of cells, and this communication was remarkably strengthened in HCC, especially with HCC malignant cells (**Figure 3A**). This finding, coupled with the result in **Figure 1D**, triggered us to study the communication between macrophages and hepatocytes or HCC malignant cells *via* receptor–ligand interactions. The results clearly showed that macrophages communicated with all the types of cells using SPP1, especially SPP1–CD44 interaction, which was not identified in tumor-adjacent tissues, suggesting the role of macrophage-derived SPP1 in the progress of HCC (**Figure 3B**). SPP1 was a well-studied oncogene in HCC, and previous studies were primarily concerned with the role of tumor cell-intrinsic SPP1. The most recent study indicated that tumor cell-intrinsic SPP1 could promote macrophages to M2-like tumor-associated macrophages (TAMs) by mediating the crosstalk between HCC malignant cells and macrophages. Our findings further noted there may be a mutually reinforcing cycle.

To further verify the result from **Figure 1D**, we analyzed the proportion of macrophages (clusters 2, 4, 5, and 26) in tumor-adjacent and HCC groups. The results observed a significant enrichment of macrophages in tumors compared with tumor-adjacent tissues (**Figure 3C**). Then, we attempted to distinguish M1 and M2 macrophages from clusters 2, 4, 5, and 26. We found that the marker genes of M1 macrophages (FCGR3A) and M2 macrophages (CD163) were all expressed in clusters 2, 4, 5, and 26, in accordance with a previous report (18). Therefore, we analyzed and calculated M1 and M2 polarization scores of clusters 2, 4, 5, and 26 using macrophage polarization-related gene sets (**Table S2**) (18). It was demonstrated that all the clusters of macrophages have an M2-like phenotype (**Figures 3D, E**).

### Gene Ontology and Kyoto Encyclopedia of Genes and Genomes Pathway Analysis and Protein–Protein Interaction Network Construction

**Figure 4:**
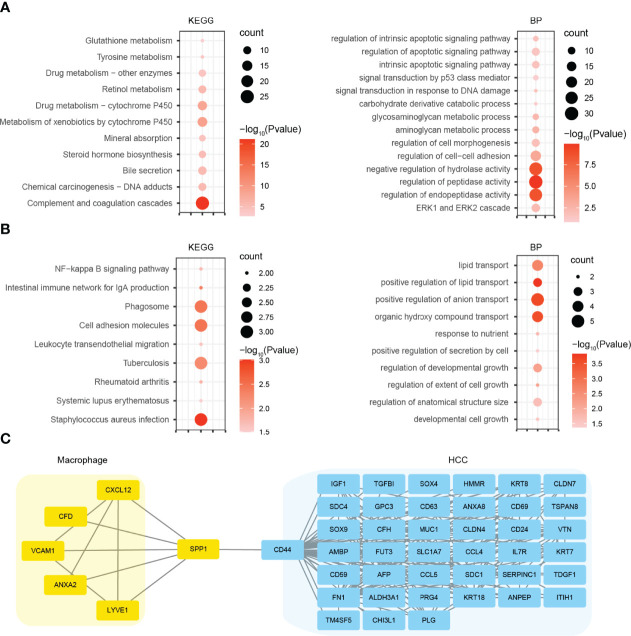
GO and KEGG pathway analyses and PPI network construction. **(A)** KEGG and GO BP analyses using the DEGs of HCC malignant cells. **(B)** KEGG and GO BP analyses using the DEGs of macrophages. **(C)** PPI network using the genes directly interacting with SPP1 or CD44. GO, Gene Ontology; KEGG, Kyoto Encyclopedia of Genes and Genomes; PPI, protein–protein interaction; BP, biological process; DEGs, differentially expressed genes; HCC, hepatocellular carcinoma.

For a deeper insight into the biological alteration of HCC malignant cells and macrophages, we performed GO and KEGG pathway enrichment analyses. KEGG analysis showed that the DEGs in HCC malignant cells were mainly enriched in metabolism-related pathways, including glutathione metabolism, tyrosine metabolism, and retinol metabolism (**Figure 4A**). GO analysis indicated that the DEGs significantly enriched in metabolic process, as well as apoptosis signaling pathways (**Figure 4A**). For macrophages, we found that the DEGs were mainly enriched in cell adhesion molecules, phagosome, and immune-related pathways by KEGG analysis, and lipid transport and growth-related process (**Figure 4B**). Furthermore, we constructed the PPI networks using the DEGs in HCC malignant cells and macrophages. Coincidentally, SPP1 and CD44 were both the top hub genes in their respective PPI network. So we merged these two PPI networks using the genes directly interacting with SPP1 or CD44 (**Figure 4C**). The new network indicated many potential signaling axes that mediate the crosstalk between HCC malignant cells and macrophages, which need further validation.

### Determination of the Expression and Prognostic Role of SPP1 and CD44 in Hepatocellular Carcinoma

**Figure 5:**
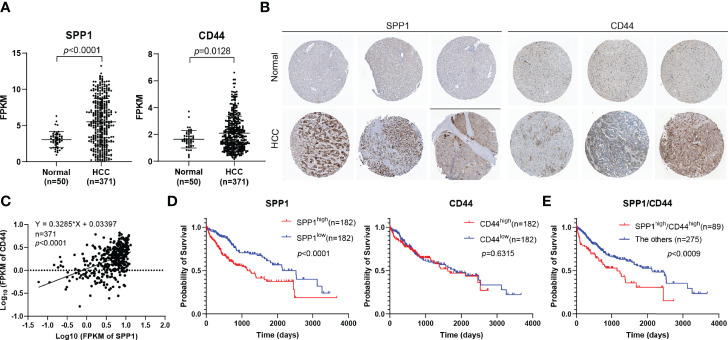
Determination of the expression and prognostic role of SPP1 and CD44 in HCC. **(A)** The mRNA expression of SPP1 and CD44 in HCC from the TCGA-LIHC database. **(B)** IHC staining of SPP1 and CD44 in normal and HCC tissues from HPA database. **(C)** The correlation of the expression of SPP1 and CD44 in HCC (n = 424) from TCGA-LIHC database. **(D, E)** Kaplan–Meier survival curve of SPP1 and CD44 in HCC from TCGA-LIHC database. HCC, hepatocellular carcinoma; TCGA-LIHC, The Cancer Genome Atlas Liver Hepatocellular Carcinoma; IHC, immunohistochemistry; HPA, Human Protein Atlas.

According to the findings above, we further analyzed the expression of SPP1 and CD44 in HCC using TCGA-LIHC database. The results suggested that SPP1 and CD44 expression was significantly upregulated in HCC tissues than in normal tissues (**Figure 5A**). Then, the expression of SPP1 and CD44 at the protein level was also analyzed using the HPA database. The immunohistochemistry (IHC) results showed that the protein expression of SPP1 and CD44 in HCC was significantly higher than that in normal tissues (**Figure 5B**). We were surprised to find a close association between the expression of SPP1 and CD44, suggesting the potential regulation relationship between SPP1 and CD44, which needs to be further verified (**Figure 5C**). Furthermore, we also downloaded the survival data from TCGA-LIHC dataset. By combining the expression data and survival data, we found that high expression of SPP1 significantly indicated poor prognosis in HCC, but high expression of CD44 was not (**Figure 5D**). Finally, we divided the patients into SPP1^high^/CD44^high^ (n = 89) and the other group (n = 275). The survival result showed the worse prognosis of patients with dual high expression of SPP1 and CD44, indicating the pro-tumor role of the SPP1/CD44 axis in the progress of HCC (**Figure 5E**).

## Discussion

Despite the advances in surveillance and treatment strategies, the clinical outcomes of HCC remain unsatisfactory, mainly because of the lack of in-depth understanding of heterogeneity (27). Hence, we focused on the exploration of the intratumoral heterogeneity in HCC. Here, we characterized the ecosystems of tumor-adjacent and HCC tissues by bioinformatics analysis of single-cell transcriptomic data, revealing a distinct immune ecosystem in HCC. We found that the proportions of pDCs and NK cells were decreased and the proportion of macrophages was increased in the immune component.

Immunotherapy is a promising approach that stimulates immune cells to enhance their anticancer activity (28). Crosstalk among different cell types plays a crucial part in the efficacy of immunotherapy. Therefore, CellPhoneDB was used to explore cell–cell communications. We found that there was obviously an interaction between HCC malignant cells and macrophages, which may compromise antitumor immunity. Liver macrophages (Mϕs) mainly consist of resident Kupffer cells and MoMϕs. The TME of HCC regulates the polarization of macrophages, resulting in M2-like macrophages with immunosuppressive properties. In this study, we tried to identify the phenotype of macrophages (clusters 2, 4, 5, and 26). We found the marker genes of M1 macrophages (FCGR3A) and M2 macrophages (CD163) were all expressed in clusters 2, 4, 5, and 26, in accordance with a previous report (18). Therefore, we analyzed clusters 2, 4, 5, and 26 using macrophage polarization-related gene sets. It was demonstrated that all the clusters of macrophages have an M2-like phenotype.

Using CellPhoneDB database, we also identified multiple ligand–receptor interactions mediating cell crosstalk, including FcRn complex–albumin (ALB) and SPP1–CD44 between macrophages and HCC malignant cells. FcRn complex–ALB interaction exists in normal liver tissues. However, the strength of interaction was enhanced in HCC. It was demonstrated that hepatocytes used FcRn complex receptors to bind ALB to maintain its normal growth and metabolism. With the progress of HCC, malignant cells consumed a large amount of nutrients for proliferation or invasion, mainly by binding more ALB (29). The upregulated expression of FcRn was identified in a number of cancers, suggesting the importance of ALB recruitment driven by FcRn (30) and the recycling and transcytosis of ALB regulated by FcRn (31, 32). In addition to FcRn complex–ALB interaction, we found that macrophages communicated with all the types of cells using SPP1, especially SPP1–CD44 interaction, which was not identified in normal tissues, suggesting the role of macrophage-derived SPP1 in the progress of HCC (**Figure 3B**). SPP1 was a well-studied oncogene in HCC, and previous studies were primarily concerned with the role of tumor cell-intrinsic SPP1. The most recent study indicated that HCC cells could secrete SPP1 into TME and bind to CD44 of macrophages, resulting in M2-phenotype TAM polarization of macrophages. Our findings further noted that there may be a mutually reinforcing cycle.

In conclusion, our study characterizes the heterogeneity of the tumor ecosystem between tumor-adjacent and HCC tissues, especially the crosstalk between immune cells and HCC malignant cells. Moreover, we identify the SPP1–CD44 axis as a unique interaction between macrophages and HCC malignant cells. Our comprehensive portrait of cell communication patterns over the HCC ecosystem reveals further insights into immune infiltration and more effective therapeutic targets for immunotherapies in patients with HCC.

## Data Availability Statement

The datasets presented in this study can be found in online repositories. The names of the repository/repositories and accession number(s) can be found in the article/**Supplementary Material**.

## Author Contributions

JQ conceived and supervised the study. YL and LZ collected and analyzed the data. SW and JQ wrote the manuscript. XJ and JQ revised the manuscript. All authors read and approved the manuscript and agreed to be accountable for all aspects of the research in ensuring that the accuracy or integrity of any part of the work is appropriately investigated and resolved.

## Conflict of Interest

The authors declare that the research was conducted in the absence of any commercial or financial relationships that could be construed as a potential conflict of interest.

## Publisher’s Note

All claims expressed in this article are solely those of the authors and do not necessarily represent those of their affiliated organizations, or those of the publisher, the editors and the reviewers. Any product that may be evaluated in this article, or claim that may be made by its manufacturer, is not guaranteed or endorsed by the publisher.

## Supplementary Material

Supplementary Figure 1 Overview of the cell clusters based on scRNA-seq data using total cells 23,225 cells from tumor-adjacent tissues (control, n = 8), and 22,677 cells from HCC tissues (n = 10).

Supplementary Figure 2 DEGs in non-immune cells. **(A)** Endothelial cells. **(B)** Epithelial cells. **(C)** HCC malignant cells. **(D)** HSC cells.

Supplementary Table 1 The results of cell cross-talk from CellPhoneDB.

Supplementary Table 2 Signature related gene sets used in macrophage analysis.
