# Supplementary figures and images for: Single-Cell Transcriptomic Analysis Reveals Macrophage–Tumor Crosstalk in Hepatocellular Carcinoma
Source: Front Immunol. 2022 Jul 25;13:955390. doi: 10.3389/fimmu.2022.955390 (PMC9359093; doi:10.3389/fimmu.2022.955390)

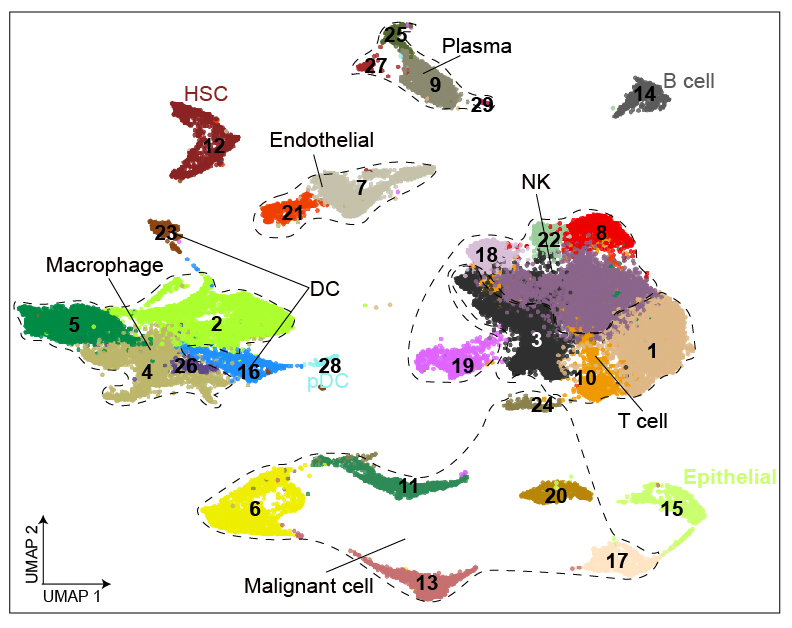

Supplement: Supplementary Figure 1 — Overview of the cell clusters based on scRNA-seq data using total cells 23,225 cells from tumor-adjacent tissues (control, n = 8), and 22,677 cells from HCC tissues (n = 10). [file Image_1.jpeg]

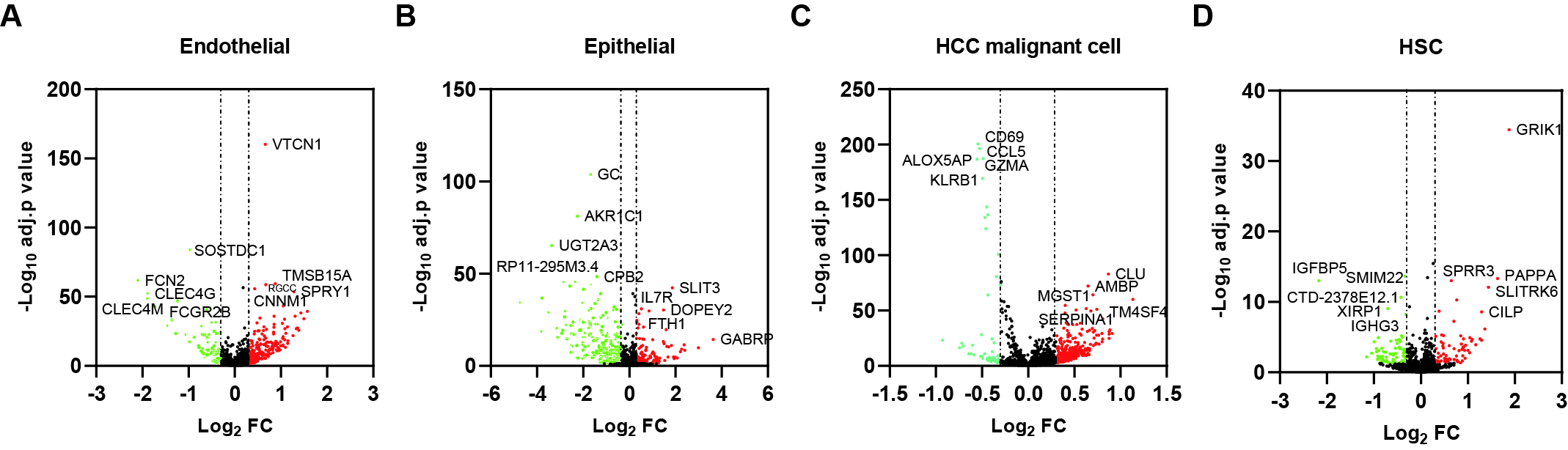

Supplement: Supplementary Figure 2 — DEGs in non-immune cells. (A) Endothelial cells. (B) Epithelial cells. (C) HCC malignant cells. (D) HSC cells. [file Image_2.jpeg]
