# Supplementary material for: Single-Cell Transcriptomic Analysis Reveals Macrophage–Tumor Crosstalk in Hepatocellular Carcinoma
Source: Front Immunol. 2022 Jul 25;13:955390. doi: 10.3389/fimmu.2022.955390 (PMC9359093; doi:10.3389/fimmu.2022.955390)
Supplement: Supplementary file 5 [file DataSheet_1.docx]

**Supplementary materials and methods**

This supplementary document described the more detailed content, including the processing and generation process of each intermediate data, results and related codes.

The link is “https://www.jianguoyun.com/p/DUD7TkIQxp3MChja2MAEIAA”

**scRNA-Seq Data Processing (including Fig.1 and Fig.S1 production)**

The scRNA-Seq Dataset of GSE149614 was obtained from the Gene Expression Omnibus (GEO) database (<https://ncbi.nlm.nih.gov/geo/query/acc.cgi?acc=GSE149614>), and the processed count matrix data (GSE149614_HCC.scRNAseq.S71915.count.txt) was downloaded for further analysis.

This data was processed and analyzed according to the following steps: data importing, batch effect removing, normalization, dimension reduction by PCA, Louvain-based clustering, cluster-specific gene analysis. In particular, after clustering, the cell distribution (Fig.1A and Fig.S1) was visualized by UMAP. The annotated R code for these procedures and figure production was saved at “1. scRNA-seq-clustering-analysis-procedures.R”.

The obtained Seurat object, including the raw count data, normalized data, PCs (dim=30), sample annotation, gene annotation, cell clusters, UMAP coordinate, was saved as “2. GSE149614_Seurat.RData”.

The R code for counting cell number info of each cluster was saved at “3. cell-number-counting.R”. The generated result sheet was saved as “4. Cell numbers in each cluster.xlsx”, and then, the Fig.1B was drawn from this by Graphpad 8.0. The original GraphPad statistical file was saved as “5. Fig.1B.pzfx”.

By consulting the cell type signature summarized in Sun’s paper (Single-cell landscape of the ecosystem in early-relapse hepatocellular carcinoma, Cell, 2021), we annotated the cell clusters obtained in our project. Finally, 11 cell types were identified among the 30 clusters, including 4 types of non-immune cells and 7 types of immune cells (Fig.1C). Non-immune cells were mainly composed of endothelial cells (Es; CDH5, SPARC, TM4SF1 and INSR), hepatic stellate cells (HSCs; RGS5, COL1A1, ACTA2 and PDGFRB), apparently normal epithelial cells (KRT18, KRT19 and EPCAM) and hepatocyte or HCC malignant cells (Fig.1C). Immune cells primarily consisted of macrophage (LYZ, AIF1, HLA-DRA, CD163, CD68 and FOLR2), DCs (LYZ, AIF1, HLA-DRA and IDO1), plasmacytoid DCs (pDCs; GZMB, TCF4 and TCL1A), natural killer (NK) cells (KLRD1, KLRF1, GNLY and B3GNT7), T cells (IL7R and ITM2A), B cells (MS4A1, BANK1, CD79A and TNFRSF13C) and plasma cells (IGLL1, MZB1 and SSR4). The heatmap production code for Fig.1C was saved at “6. heatmap.R”.

The proportions of each cell type in tumor-adjacent tissues (n=8) and HCC (n=10) were calculated using the data from “4. Cell numbers in each cluster.xlsx”, and shown in Fig.1D, which was created by Graphpad 8.0 saved as “7. Fig.1D.pzfx”.

**Deferentially Expressed Genes in** **Specified cell types (Fig.2 and Fig.S2)**

The differential genes between tumor and non-tumor tissues for each cell type were analysis by limma package, and the R code for the analysis was saved as “8. tumor-normal_differential-gene.R”. The analysis results were included in the folder named “9. The DEGs in each cell type”. The volcano plots (Fig.2 and Fig.S2) were created by Graphpad 8.0 saved as “10. Fig.2.pzfx”.

**Cell Crosstalk Analysis (Fig.3A, 3B, 3C and 3D)**

The cell-cell communication was analyzed according to CellphoneDB method (<https://www.cellphonedb.org/>). The data was included in the folder “11. Cell-cell communication”, including 4 input files and two output files, the bash code for running this software was save at “12. running-cellphoneDB.sh”. Fig.3A was created by Excel saved as “13. Normal_significant_means.xlsx” and “14. Tumor_significant_means.xlsx”.

The data of ligand-receptor interaction between cell types was from the output files in the folder “11. Cell-cell communication”, and the R code for figure creation (Fig.3B) was saved at “15. bubble.R”.

The proportions of macrophage (cluster 2, 4, 5, and 26) in tumor-adjacent and HCC groups were analyzed using the data from “4. Cell numbers in each cluster.xlsx”. The result (Fig.3C) was created by Graphpad 8.0 saved as “16. Fig.3C.pzfx”.

The specific genes of M1/M2 macrophage were listed in “17. Signature related gene sets used in macrophage analysis.xlsx”. The expression of these genes in cluster 2, 4, 5, and 26 was shown in Fig.3D, which was created by Excel. The original data was saved in “18. Cluster_Specific_Gene.xlsx”.

**Definition of macrophage scores (Fig.3E)**

The activity propensity of each cell in clusters 2, 4, 5, 26 was analyzed using the GSVA package and the R code for GSVA analysis was saved at “19. GSVA-analysis.R”. The result was shown in Fig.3E, which was created by Graphpad 8.0 saved as “20. Fig.3E.pzfx”.

**GO and KEGG Pathway Functional Enrichment Analysis (Fig.4A and 4B)**

The data of DEGs in HCC and macrophage was from “9. The DEGs in each cell type”. The gene set enrichment analysis R code was saved at “21. clusterProfiler.R” and the analysis results were saved in fold “22. annotation”. The figures creation (Fig.4A and 4B) code was saved at “15. bubble.R”.

**Protein-protein interaction (PPI) Network Construction (Fig.4C)**

The data of DEGs in HCC and macrophage was from “9. The DEGs in each cell type”. The DEGs were mapped in the STRING database (http://strin g-db.org) to assess protein-protein interaction (PPI) within HCC tissues. The PPI network (Fig.4C) was constructed using Cytoscape software (version 3.6.0) and the original data was saved as “23. ppi.cys”.

**Validation of differential expression of selected genes (Fig.5A, 5B and 5C)**

The TCGA-LICH data was obtained from UCSC Xena (https://xenabrowser.net/datapages/?cohort=GDC%20TCGA%20Liver%20Cancer%20(LIHC)&removeHub=https%3A%2F%2Fxena.treehouse.gi.ucsc.edu%3A443), and the detailed information was included in the folder “24. TCGA-LICH datasets”. The figure (Fig.5A) was created by Graphpad 8.0 saved as “25. Fig.5A.pzfx”.

The Human Protein Atlas (HPA) database (<https://www.proteinatlas.org/>, for protein expression) were used in our study (Fig.5B).

The correlation of the expression of SPP1 and CD44 in HCC was analyzed and visualized (Fig.5C) by Graphpad 8.0, which was saved as “26. Fig.5C.pzfx”

**Survival analysis (Fig.5D and 5E)**

HCC patient’s gene expression data and the survival data were included in the folder “24. TCGA-LICH datasets”. Kaplan-Meier survival curves were visualized (Fig.5D and 5E) by Graphpad 8.0 and survival difference between groups was test by log-rank test. The original GraphPad statistical file was saved as “27. Fig.5D and 5E.pzfx”.
